# Nanoscopic Interfacial Hydrogel Viscoelasticity Revealed from Comparison of Macroscopic and Microscopic Rheology

**DOI:** 10.1021/acs.nanolett.3c04884

**Published:** 2024-04-09

**Authors:** Robert F. Schmidt, Henrik Kiefer, Robert Dalgliesh, Michael Gradzielski, Roland R. Netz

**Affiliations:** † Stranski-Laboratorium für Physikalische und Theoretische Chemie, Technische Universität Berlin, Strasse des 17. Juni 124, 10623 Berlin, Germany; ‡ Fachbereich Physik, Freie Universität Berlin, Arnimallee 14, 14195 Berlin, Germany; § STFC, ISIS, Rutherford Appleton Laboratory, Chilton, Oxfordshire OX11 0QX, United Kingdom

**Keywords:** Hydrogels, nanoparticles, diffusion, power-law rheology, viscoelasticity, interfacial rheology

## Abstract

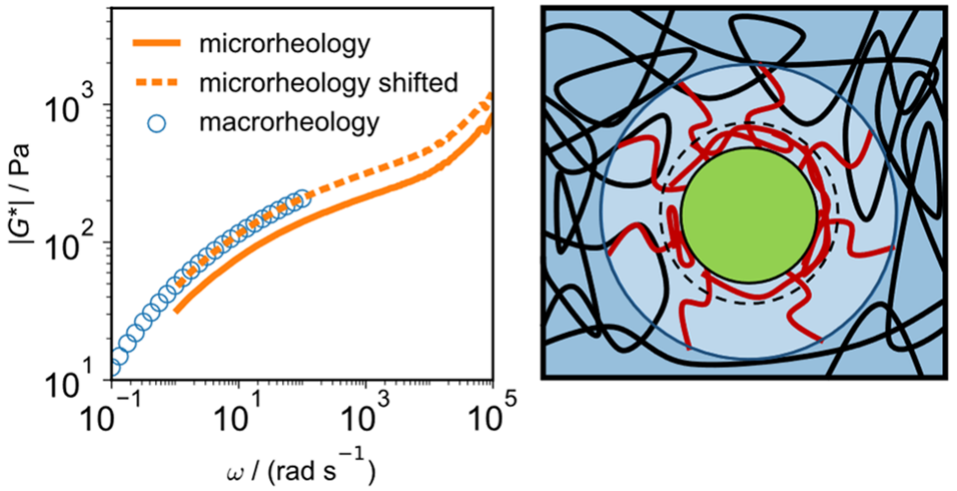

Deviations between macrorheological and particle-based microrheological measurements are often considered to be a nuisance and neglected. We study aqueous poly(ethylene oxide) (PEO) hydrogels for varying PEO concentrations and chain lengths that contain microscopic tracer particles and show that these deviations reveal the nanoscopic viscoelastic properties of the particle–hydrogel interface. Based on the transient Stokes equation, we first demonstrate that the deviations are not due to finite particle radius, compressibility, or surface-slip effects. Small-angle neutron scattering rules out hydrogel heterogeneities. Instead, we show that a generalized Stokes–Einstein relation, accounting for an interfacial shell around tracers with viscoelastic properties that deviate from bulk, consistently explains our macrorheological and microrheological measurements. The extracted shell diameter is comparable to the PEO end-to-end distance, indicating the importance of dangling chain ends. Our methodology reveals the nanoscopic interfacial rheology of hydrogels and is applicable to different kinds of viscoelastic fluids and particles.

Soft matter materials are generally viscoelastic, meaning that they exhibit a viscous, elastic, or intermediate response to external perturbations, depending on the response time. In macrorheology, a macroscopic amount of material is deformed by applying strain or stress, and the resulting force or displacement response is measured, respectively.^1^ A common macrorheological technique is oscillatory shear rheology, where the sample is subject to an oscillating shear strain and the resulting oscillating shear stress is measured, yielding the complex modulus G* as a function of frequency. In contrast, in microrheology, the viscoelastic behavior of the sample is extracted from the active or passive motion of dispersed microscopic tracer particles.^2−4^ Microrheology offers several advantages over macrorheology, such as a smaller sample volume, the ability to probe locally in spatially heterogeneous samples, and access to much higher frequencies.

Ideally, one would like to combine macro- and microrheological techniques and obtain the viscoelastic sample response over a comprehensive frequency range, for which one needs to accurately extract the viscoelastic modulus from the tracer-particle dynamics. This is accomplished by the generalized Stokes–Einstein relation (GSER), which connects the macroscopic sample viscoelasticity to the frequency-dependent friction experienced by a tracer particle.^5,6^ Because of its importance for the understanding of soft-matter dynamics, the GSER has been the subject of numerous experimental and theoretical investigations.^7−15^ Several studies have compared macro- and microrheological measurements on the same sample.^5,16−19^ Using the GSER for the conversion of the microrheology data, the reported agreement of the complex modulus G* in the overlap frequency range is typically rather good; however, upon closer inspection, it is evident that macro- and microrheological data exhibit systematic deviations, in the sense that microrheology experiments show enhanced or reduced viscoelastic response compared to macrorheology, depending on specificities of the sample and the tracer particles.^16,17,20^

This is where our paper comes in: We show that the experimentally determined deviations between macro- and microrheological spectra for a synthetic polymeric hydrogel reveal the effect of polymer–particle interactions on the effective hydrogel viscoelasticity around the probe particles. We employ semidilute aqueous solutions of linear poly(ethylene oxide) (PEO) polymers, which are hydrogels with physical cross-links due to polymer chain entanglements^21−24^ and constitute ideal model systems because of their simple structure and reproducible properties.^16,18,25−28^ We tune the PEO hydrogel viscoelasticity by changing both the PEO concentration and chain length.

The GSER has been argued to hold for homogeneous and incompressible samples^5,6^ and in the absence of slip on the tracer-particle surface.^29^ In fact, finite compressibility of the viscoelastic sample, slip effects and finite tracer particle size can be exactly accounted for by the solution of the transient Stokes equation for a viscoelastic fluid in spherical geomery,^30^ but does not explain the deviations between our macro- and microrheology hydrogel data, as shown below. The effect of sample inhomogeneity is more subtle: A hydrogel, i.e., a dilute entangled polymer solution, is structurally characterized by its mesh size.^31^ For tracer particles significantly larger than the mesh size, the hydrogel can be considered homogeneous on the characteristic particle length scale, and the particles probe the macroscopic hydrogel viscosity. Particles much smaller than the mesh size can diffuse through the hydrogel meshes and are subject to the solvent viscosity, unless they are strongly attracted to the polymers making up the hydrogel.^32,33^ The intermediate situation, if the particle size is of the order of the hydrogel inhomogeneity, characterized by the mesh size, constitutes an immensely complex problem.^34,35^ In our experiments, the tracer particles are significantly larger than the hydrogel mesh size, as determined from small-angle neutron scattering (SANS) measurements, so we can confidently assume that the particles probe the macroscopic hydrogel viscoelasticity. Yet, there is another effect that intrinsically differentiates macro- from microrheological data and has hitherto not been studied in detail: Any tracer-particle material will interact attractively or repulsively with the hydrogel polymer and thereby induce polymer adsorption or depletion.^36−38^ As a consequence, the effective hydrogel viscoelasticity in the vicinity of the particle surface will differ from its bulk value. By using a simple shell model for the hydrogel viscoelastic properties,^39,40^ we demonstrate in this paper that we can not only explain the commonly observed deviation between macro- and microrheological data but also derive the effective viscosity in the hydrogel interfacial layer from these deviations.

## Macrorheological Viscoelastic Spectra of PEO Solutions

**Figure 1:**
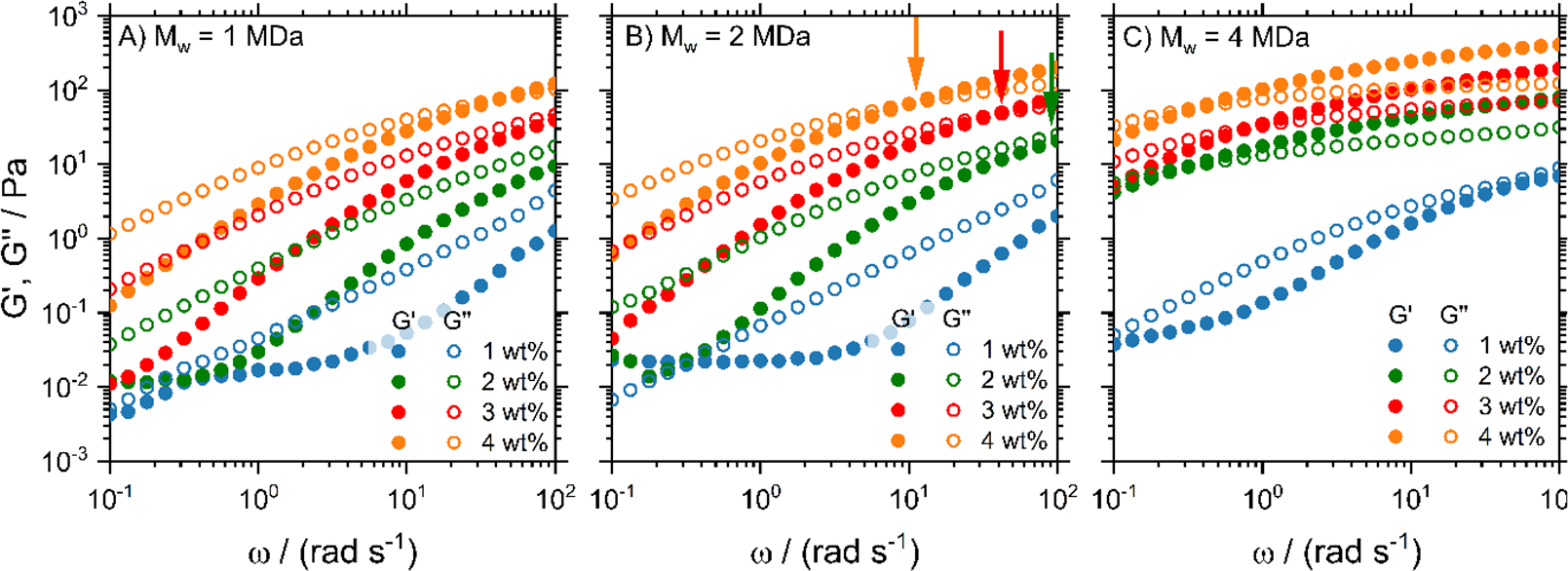
Storage (*G*′) and loss (*G*″) moduli from macrorheological oscillatory frequency sweeps for PEO solutions with different concentrations and molecular weights of (A) 1, (B) 2, and (C) 4 MDa. The vertical arrows in panel (B) indicate crossover frequency ω_0_.

Frequency sweeps on poly(ethylene oxide) (PEO) solutions, which are viscoelastic in the semidilute regime (see Supporting Information (SI) Section S1, for details), were performed for varying polymer concentration *c* and chain length (i.e., molecular weight *M*_w_ ) with a strain amplitude of γ_0_ = 5% and angular frequencies between 0.1 and 100 rad/s (see SI Sections S2 for sample preparation and S3 for experimental details). The results in Figure 1 demonstrate that the elastic *G′* and viscous *G″* moduli increase with concentration and chain length. The low-frequency plateau of *G*′ for the low-viscosity samples is a measurement artifact due to phase-angle uncertainties and expected for samples with low-torque signals.^20^ For 1 MDa PEO (Figure 1A), all samples are predominantly viscous since *G″* > *G′* for all concentrations and frequencies except for the highest concentrated 4% sample, where we see a crossover at very high frequencies. The inverse crossover frequency ω_0_ indicates a balance between entanglement and disentanglement dynamics and defines the effective relaxation time τ_0_ = 2π/ω_0_.^41^ With increasing concentration, ω_0_, indicated by arrows in Figure 1B, shifts to lower frequencies. For the 4 MDa PEO (Figure 1C), on the other hand, *G′* dominates for most concentrations and frequencies, indicating that these samples behave predominantly elastically. Our samples thus cover the full range of viscoelastic behavior. In SI Section S4 it is shown that the frequency dependence of *G′* and *G″* is well described by the fractional Maxwell model, which features power-law spectral behavior.^42^

## Microrheological Viscoelastic Spectra

**Figure 2:**
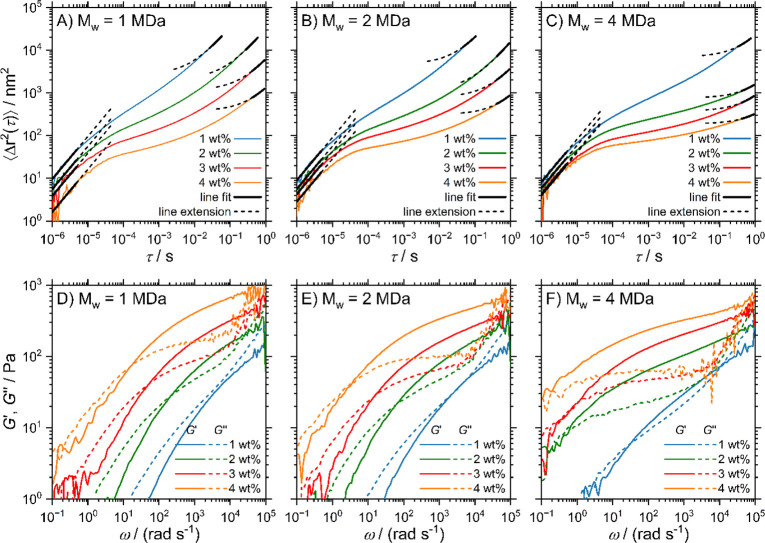
(A–C) Mean-squared displacements ⟨Δ*r*^2^(τ)⟩ and (D–F) storage (*G*′) and loss moduli (*G*″) determined using DLS microrheology on PEO solutions containing PS-109 tracer particles. The full black lines in panels (A–C) indicate asymptotic linear fits, which have been extended by one decade (broken black lines). The value of the constant in the long-time linear fits is substantial, explaining the curvature in the log–log plots.

Microrheological experiments using dynamic light scattering (DLS) were performed on the same PEO samples that contain polystyrene (PS) tracer particles with hydrodynamic diameters of 68.8 (termed PS-69), 109.3 (PS-109), and 192.0 nm (PS-192). The DLS measurements yield the intensity autocorrelation function *g*^(2)^(τ), which is converted into the mean-squared displacement (MSD) ⟨Δ*r*^2^(τ)⟩ shown in Figure 2A–C. Only the highly viscous 4 MDa samples for 3 and 4 wt % exhibit slight deviations among different spatial measurement positions caused by the long relaxation times in these systems (for details and additional data, see SI Section S5).

The MSD is related to the frequency-dependent storage and loss moduli by the generalized Stokes–Einstein relation (GSER)^5,6,25,43^
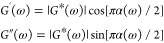
1with
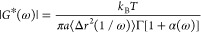
2where *k*_B_ is the Boltzmann constant, *T* the temperature, *a* the hydrodynamic tracer-particle radius, and ω the angular frequency. Here,  denotes the Gamma function. The MSDs are expressed as power laws with frequency-dependent exponent α(ω) and converted into viscoelastic moduli (see SI Sections S5 and S6).^25,43^ The results for the PS-109 samples are shown in Figure 2D–F.

**Figure 3:**
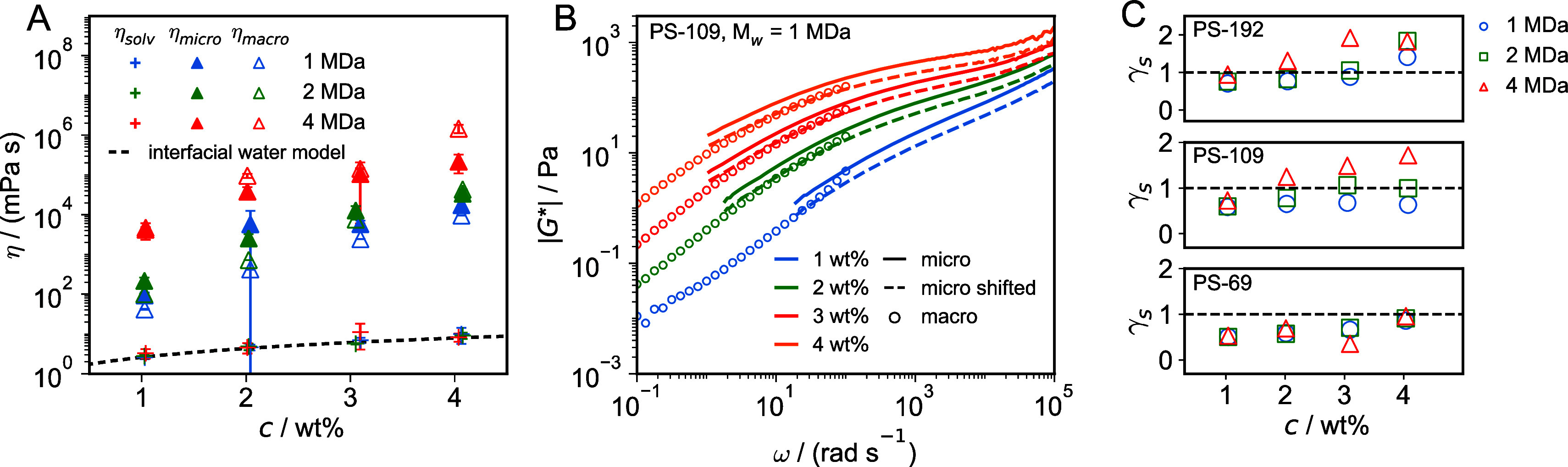
(A) Viscosities of the solvent and the hydrogel, η_solv_ and η_micro,_ determined from linear fits of the short- and long-time behavior of the MSDs extracted from microrheology in Figure 2, compared to η_macro_, determined from macrorheological steady-shear experiments. The broken line indicates the effective solvent viscosity of a polymer solution according to eq 4, which accounts for the increased viscosity of interfacial water surrounding the polymers. (B) Comparison of the viscoelastic moduli  from macro- and microrheological measurements for PS-109 tracer particles in 1 MDa PEO solutions (for the other data sets, see SI Section S8). Circles denote macrorheology, and solid lines denote microrheology results. Broken lines denote the microrheological data that is shifted by a factor γ_*s*_ to match the macrorheology data (see SI Section S8). (C) Shift factor γ_*s*_ for different tracer-particle sizes and PEO molecular weights (○, 1 MDa; □, 2 MDa; Δ, 4 MDa) as a function of PEO concentration. The black horizontal line denotes γ_*s*_ = 1, i.e., perfect agreement between macro- and microrheology.

Neglecting finite particle mass in a purely viscous liquid, the particle MSD is linear in time. Particles trapped in a purely elastic solid never leave their initial position; therefore, the MSD is constant. For viscoelastic hydrogels, three consecutive scaling regimes occur. At very short times, polymers do not influence the particle dynamics, which is determined only by the solvent viscosity,^44^ ⟨Δ*r*^2^(τ)⟩ = 6*D*_solv_τ, where *D*_solv_ is the particle diffusion coefficient in pure solvent. We determine *D*_solv_ from a fit according to ⟨Δ*r*^2^(τ)⟩ = 6*D*_solv_τ of the short-time MSD (see SI Section S7), for 10^–6^ < τ < 5 × 10^–6^ s. The solvent viscosity η_solv_ follows from the Stokes–Einstein equation *D*_solv_ = *k*_B_*T*/(6πη_solv_*a*). At intermediate times, the particles exhibit subdiffusion, ⟨Δ*r*^2^(τ)⟩ ∼ τ^α^ with 0 < α < 1, reflecting hydrogel viscoelasticity. At very long times, the MSD becomes diffusive again, ⟨Δ*r*^2^(τ)⟩ = 6*D*_micro_τ + *b*, where *D*_micro_ = *k*_B_*T*/(6πη_micro_*a*) characterizes the linear hydrogel viscosity η_micro_ and *b* is a constant shift.^18,45^ Three measurements were performed per chain length and concentration, one for each tracer-particle radius *a*. Since no significant differences were found for varying *a*, the three values of η_solv_ and η_micro_ were averaged, and the results are shown in Figure 3A.

The extracted solvent viscosity η_solv_ in Figure 3A increases with polymer concentration *c* but, expectedly, is independent of the chain length. The values for η_solv_ range from 2 to 15 mPa s and are thus significantly larger than the viscosity of pure water at 25 °C, which is η_w_ = 0.89 mPa s. In molecular dynamics simulations it was shown that the interfacial water layer at a polar surface exhibits a significantly increased water viscosity.^46^ The thickness of that interfacial layer was obtained as *d* = 0.4 nm. To explain the increase in η_solv_ with *c*, we regard each PEO polymer as being surrounded by an interfacial water layer with increased viscosity η_*i*_. We model the hydrated polymers as cylinders with radius *R*_cyl_ = (*R*_PEO_ + *d*), where *R*_PEO_ = 0.229 nm is the radius of a stretched PEO chain, estimated from the density of a PEO melt (see SI Section S9). The volume fraction of hydrated polymers is then given by
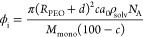
3where *c* is the polymer mass percentage, *a*_0_ = 0.356 nm is the PEO monomer length,^47^ ρ_solv_ is the water mass density, *N*_A_ is Avogadro’s constant, and *M*_mono_ = 44.05 g/mol is the molar mass of a PEO monomer. From ϕ_i_, the overall solvent viscosity follows from a simple geometric model (see SI Section S10) as
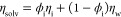
4where η_*i*_ and η_*w*_ are the viscosities of interfacial and bulk water, respectively. Using η_w_ = 0.89 mPa s and *d* = 0.4 nm, the fit of eq 4 to our experimental data (broken line in Figure 3A) yields η_i_ = (27.17 ± 0.74) mPa s, in good agreement with the simulation results.^46^ We thus conclude that the increase of the solvent viscosity from microrheology can be well explained by the increased viscosity of the interfacial water layers around the PEO.

Additionally, the hydrogel viscosity was extracted from nonoscillatory macrorheological measurements at steady shear rate γ̇ by fits to the nonlinear Cross model (see SI Section S11). Since η_macro_ is the limiting value for zero shear rate, it is the linear-response viscosity that can be compared to η_micro_ from microrheology. As evidenced in Figure 3A, η_macro_ and η_micro_ are comparable, but systematic shifts are observed, as will be discussed and explained in detail below.

## Comparison between Macro- and Microrheology

In Figure 3B we compare the absolute values of the viscoelastic modulus  from microrheology and macrorheology for tracer particle PS-109 and polymer weight *M*_w_ = 1 MDa. Deviations are quantified by a frequency-independent shift factor γ_*s*_ according to |*G*_micro,shifted_^*^| = γ_*s*_|*G*_micro_^*^| (see SI Section S8), where a value γ_*s*_ = 1 indicates the validity of the GSER. The shifted |*G*_micro,shifted_^*^|, shown in Figure 3B as broken lines, perfectly agree with the macrorheological data. Some discrepancies are observed for the samples with longer polymers, presumably due to inaccuracies of macrorheological measurements at high frequencies due to inertial effects (SI Section S3) as well as long polymeric relaxation times. In Figure 3B, γ_*s*_ is demonstrated to systematically increase with polymer concentration, while there is a much weaker and less clear dependence on tracer-particle size and chain length.

To investigate the mechanism behind the discrepancies between macro- and microrheology and the salient dependence of the shift factor γ_*s*_ on polymer concentration, we derive a generalized GSER from the transient Stokes equation around a sphere of radius *a* that includes slip on the sphere surface and compressibility in the embedding fluid. The transient Stokes equation includes a general frequency-dependent viscosity and thus correctly accounts for fluid viscoelasticity. As detailed in SI Section S12, we find no significant effects due to the finite-sphere radius for *a* below 10 μm in the experimental frequency range of 10^–1^ < ω < 10^5^ rad/s. Also, finite slip always decreases the particle friction, in contrast to the deviation between macro- and microrheology in Figure 3B, which for some experiments suggests a strong enhancement of particle friction. Thus, the GSER in eq 2, which neglects finite sphere radius, compressibility, and slip effects, is for the employed particle radii and particle types an accurate approximation of the exact solution of the transient Stokes equation derived in SI Section S12.

The GSER eq 2 furthermore assumes a homogeneous viscoelastic medium and thus neglects the hydrogel structuring on the scale of the mesh size ξ.^39,48,49^ For particle radii *a* ≫ ξ this assumption is warranted,^16,18,50^ for smaller particles deviations are expected.^51^ Since the mesh size is experimentally only indirectly accessible,^52^ it is often estimated by the polymer correlation length, ξ_SANS_, as obtained from scattering experiments.^53−56^ Depending on the PEO concentration, values of ξ_SANS_ ≈ 2–8 nm were found in our SANS measurements (see SI Section S13). These lengths favorably compare to the simple cubic-lattice estimate , where ϕ_m_ is the monomeric number density (see SI Section S14). We obtain ξ_cubic_ = 3.9 nm for 4 wt % PEO and ξ_cubic_ = 7.9 nm for 1 wt % PEO, in good agreement with our SANS measurements. Since the estimated mesh sizes are much smaller than the tracer-particle radii used, which range from diameters of 69 to 192 nm, we conclude that the hydrogels are homogeneous on the tracer-particle size and deviations between macro- and microrheology cannot plausibly be explained by inhomogeneity effects in the bulk hydrogel.

**Figure 4:**
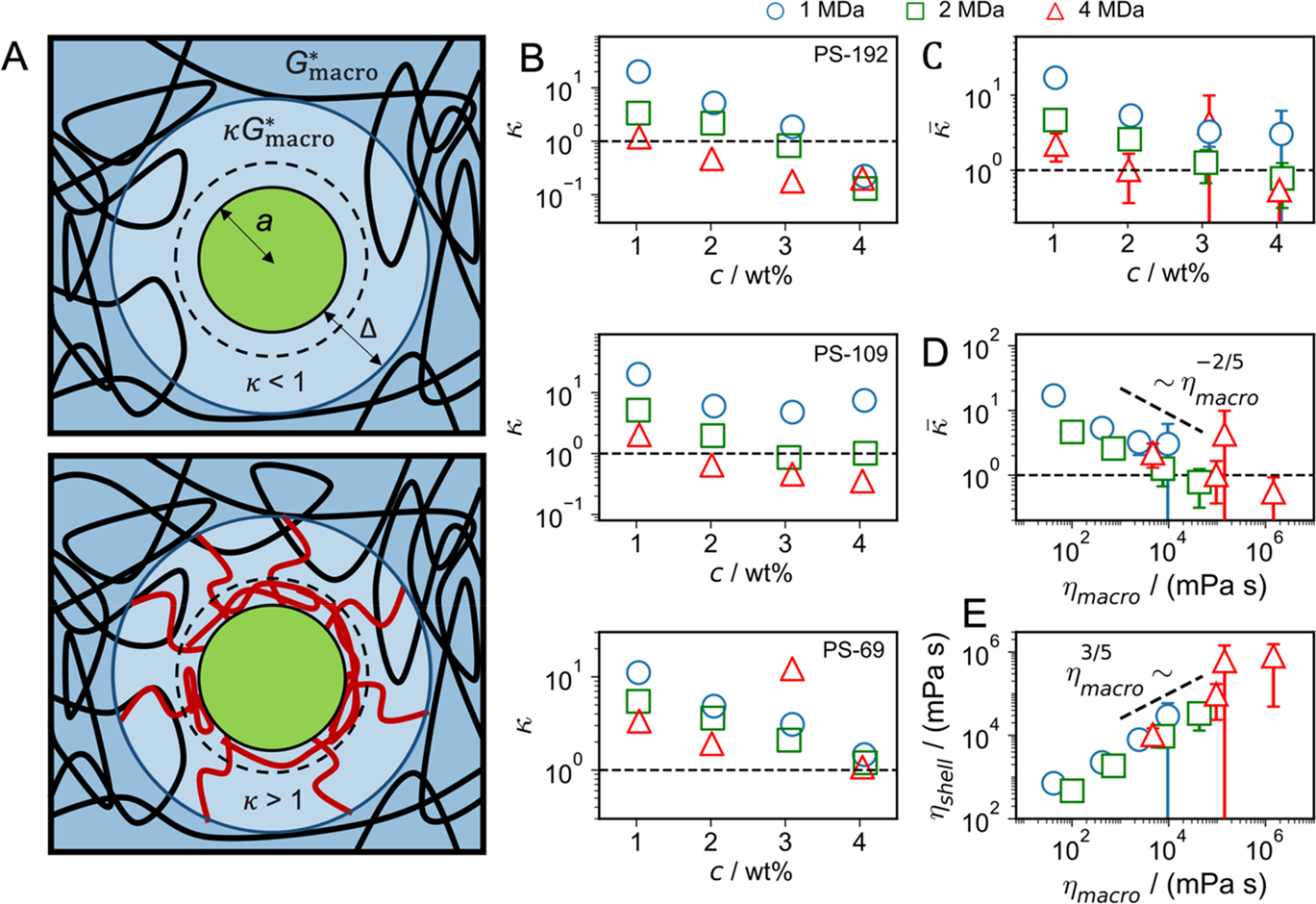
(A) Sketch of a tracer particle in a PEO hydrogel. Particle–PEO interactions produce a depletion (top) or adsorption layer (bottom), indicated by broken circles, within which the PEO density differs from the bulk. Consequently, the viscoelastic polymer response *G*_shell_^*^(ω) deviates from the bulk spectrum *G*_macro_^*^(ω) within a shell of thickness Δ (indicated by solid circles). The viscoelastic shell thickness Δ in the adsorption case (bottom) is dominated by dangling adsorbed chains (shown in red) and therefore is larger than the adsorption layer thickness. (B) Ratio of shell and bulk viscoelasticity κ = *G*_shell_^*^(ω)/*G*_macro_^*^(ω), which follows from the shift factor γ_*s*_ in Figure 3C, as a function of polymer concentration for different tracer-particle radii and PEO molecular weights. Fit errors are much smaller than the symbol size. (C, D) Interfacial viscoelastic enhancement factor averaged over the results for different tracer radii in panel B, κ̅, plotted as a function of (C) the polymer concentration and (D) the bulk viscosity η_macro_. Vertical bars indicate the standard deviation of the average over the tracer particle radii and are only shown if larger than the symbol size. (E) Interfacial shell viscosity η_shell_ = κ̅η_macro_ in dependence of bulk viscosity η_macro_. Power laws are added as guides for the eye.

We therefore consider an alternative mechanism for the GSER violation. The GSER assumes the hydrogel around the tracer particles to be entirely described by the bulk modulus *G*_macro_^*^(ω), but due to perturbations of the hydrogel around the particles, a shell with a thickness Δ and a different modulus *G*_shell_^*^(ω) will in general be present around tracer particles. As illustrated in Figure 4A, the shell within which the modulus differs from the bulk will, in general, have a different thickness Δ than the layer within which the polymer density differs from the bulk value, indicated by a broken circle.

The particle–polymer interactions can be repulsive or attractive and induce depletion^56−62^ (upper scheme) or adsorption layers^37,63−65^ (lower scheme), respectively. For depletion, one expects a shell with a reduced modulus, which would lead to a finite slip; for adsorption, one expects an increased shell viscoelastic modulus. To reduce the number of free variables in our shell model, we assume that the shell modulus is related to *G*_macro_^*^(ω) by a frequency-independent factor according to *G*_shell_^*^(ω) = κ*G*_macro_^*^(ω). The modified GSER for such a shell model has been derived from the Stokes equation and reads^39^
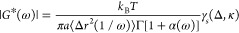
5the explicit form of the correction factor γ_*s*_(Δ, κ) is given in SI Section S15. If Δ = 0 or κ = 1 one has γ_*s*_(Δ, κ) = 1 and eq 5 converges to eq 2. Alternatively, our data could be rationalized by a modified effective tracer radius,^65−68^ but we argue that a decreased shell viscoelastic response is a more physical model than a decreased effective tracer radius (see SI Section S16). An additional horizontal shift of the microrheology data further improves the agreement with the macrorheology data, as shown in SI Section S17. Such a frequency shift suggests a modified viscoelastic relaxation time in the shell around the tracer particles, which is neglected by the modified GSER in eq 5. The parameters Δ and κ cannot be simultaneously determined from the experimentally measured γ_*s*_ values in Figure 3C, as explained in SI Section S18. By analysis of the deviation between macro- and microrheological data, we find that the shell thickness Δ is linearly related to the polymer end-to-end distance *R*_e_^ideal^, which suggests that the viscoelastic perturbation in the interfacial shell is transmitted by polymers that adsorb to the particle surface and dangle into solution, in line with literature results for the hydrodynamic radius of adsorbed polymer layers.^37,69−78^ We therefore take Δ proportional to *R*_e_^ideal^ and determine κ by the inversion of γ_*s*_(Δ, κ) for each experiment. The proportionality constant between Δ and *R*_e_^ideal^ is assumed identical for all systems and chosen as the minimal value that describes all experimental γ_*s*_ values, see SI Section S18 for details. We obtain Δ = 3/5 *R*_e_^ideal^, where the values of *R*_e_^ideal^ are given in SI Section S1.

In Figure 4B, the results for κ are shown to range between 0.1 and 20 and to generally decrease with increasing polymer concentration with a smaller dependence on particle size (see SI Section S19). We therefore average over different particle radii; the resulting average κ̅ in Figure 4C is shown to decrease with concentration and reaches κ̅ ≈ 1 for high concentration. This means that the effect of the adsorbed polymer chains on the rescaled viscoelastic modulus in the interfacial shell diminishes with increasing bulk polymer concentration, in line with the fact that the relative increase of polymer concentration in the adsorbed surface layer also decreases with increasing bulk polymer concentration.^56,79−82^ Also, κ̅ in Figure 4C decreases with increasing polymer chain length, which is plausible since the slowing down of the shell dynamics due to adsorbed polymer chains becomes less important compared to the slowing down due the hindered reptation as the polymer chains become longer.^37,83,84^ To investigate the relation between the interfacial-shell and the bulk viscosity, we plot in Figure 4D the shell/bulk modulus ratio κ̅ versus the bulk viscosity η_macro_. In this scaling plot an approximate data collapse between different polymer chain lengths occurs, and we see that the relative increase of the viscosity in the interfacial shell decreases significantly and almost universally with bulk viscosity η_macro_. Clearly, we expect the relation between κ̅ and η_macro_ to depend on the surface material, which we did not vary in the current study. The added straight line is merely meant as guide to the eye and not as proof of a power law. In Figure 4E we show the interfacial shell viscosity η_shell_ = κ̅η_macro_ as a function of the hydrogel bulk viscosity η_macro_, which demonstrates that the shell viscosity increases dramatically with increasing bulk viscosity. Although the experimental data is scarce at the highest bulk viscosities, we presume that the shell viscosity η_shell_ increases linearly with the bulk viscosity η_macro_ for η_macro_ > 10^5^ mPa s, so that η_shell_ is never smaller than η_macro_. This reflects that the polymers adsorb onto the particles, and therefore the polymer density is increased close to the particle surface.

## Conclusions

We demonstrate that the GSER is an accurate theoretical model to extract viscoelastic properties from microrheology and that observed deviations between macro- and microrheology data can be explained by interfacial effects in a shell around the tracer particles. The shell thickness is proportional to the polymer end-to-end distance and thus significantly larger than the structural adsorption layer thickness measured in scattering experiments,^64,85,86^ which reflects the importance of chain ends that dangle into the solution for the rheological properties around the particles.^37,69,70^ This not only reconciles macro- and microrheological measurements but also gives insights into the interfacial viscoelastic behavior of hydrogels and polymer solutions. Our methods are general and can be applied to more complex viscoelastic fluids and particles to investigate their interfacial rheological properties. In the future, it would be desirable to extract modified relaxation time scales and the detailed frequency-dependent viscoelastic response in the shell around the tracer particles; for this, experiments over extended frequencies would have to be performed.

## Data Availability

The data that supports the findings of this study are available from the corresponding author upon reasonable request.

## Supporting Information Available

S1: Scaling relations of PEO solutions. S2: Materials and sample preparation. S3: Macrorheology experimental details. S4: Fractional Maxwell model. S5: Microrheology experimental details. S6: Determination of the frequency-dependent power law exponent α(ω). S7: Determination of η_solv_ and η_micro_ from microrheology. S8: Determination of the shift factor. S9: Estimation of molecular PEO radius. S10: Model for effective solvent viscosity. S11: Steady-shear experiments. S12: Transient and compressibility effects in PEO solutions. S13: Small-angle neutron scattering of PEO solutions. S14: Mesh size of a cubic polymer network. S15: Derivation of the shell-model GSER. S16: Effective tracer radii from experimental shifts. S17: Additional horizontal shift of microrheology viscoelastic spectra. S18: Adjusting the dynamic moduli data using the shell model. S19: Dependence of shell modulus on tracer size. S20: Specification of particles. S21: Amplitude sweep results. (PDF)

## ABBREVIATIONS

PEO: poly(ethylene oxide)
GSER: generalized Stokes–Einstein relation
DLS: dynamic light scattering
PS: polystyrene
MSD: mean-squared displacement
SANS: small-angle neutron scattering
SI: Supporting Information
